# Differentially expressed chaperone genes reveal a stress response required for unidirectional regeneration in the basal chordate *Ciona*

**DOI:** 10.1186/s12915-023-01633-y

**Published:** 2023-06-26

**Authors:** William R. Jeffery, Bo Li, Mandy Ng, Lianwei Li, Špela Gorički, Li Ma

**Affiliations:** 1 Department of Biology, University of Maryland, College Park, MD 20742 USA; 2 Marine Biological Laboratory, Woods Hole, MA 02543 USA; 3 Station Biologique, 29680 Roscoff, France; 4 Kunming Institute of Zoology, Chinese Academy of Sciences, Kunming, 650223 China; 5 Kunming College of Life Science, University of Chinese Academy of Sciences, Beijing, 100049 China; 6 Scriptorium Biologorum, 9000 Murska Sobota, Slovenia

**Keywords:** *Ciona intestinalis*, Basal chordate, Branchial sac transcriptome, Regeneration, Stress response, HSP70 chaperone system

## Abstract

**Background:**

Unidirectional regeneration in the basal chordate *Ciona intestinalis* involves the proliferation of adult stem cells residing in the branchial sac vasculature and the migration of progenitor cells to the site of distal injury. However, after the *Ciona* body is bisected, regeneration occurs in the proximal but not in the distal fragments, even if the latter include a part of the branchial sac with stem cells. A transcriptome was sequenced and assembled from the isolated branchial sacs of regenerating animals, and the information was used to provide insights into the absence of regeneration in distal body fragments.

**Results:**

We identified 1149 differentially expressed genes, which were separated into two major modules by weighted gene correlation network analysis, one consisting of mostly upregulated genes correlated with regeneration and the other consisting of only downregulated genes associated with metabolism and homeostatic processes. The *hsp70*, *dnaJb4*, and *bag3* genes were among the highest upregulated genes and were predicted to interact in an HSP70 chaperone system. The upregulation of HSP70 chaperone genes was verified and their expression confirmed in BS vasculature cells previously identified as stem and progenitor cells. siRNA-mediated gene knockdown showed that *hsp70* and *dnaJb4,* but not *bag3,* are required for progenitor cell targeting and distal regeneration. However, neither *hsp70* nor *dnaJb4* were strongly expressed in the branchial sac vasculature of distal fragments, implying the absence of a stress response. Heat shock treatment of distal body fragments activated *hsp70* and *dnaJb4* expression indicative of a stress response, induced cell proliferation in branchial sac vasculature cells, and promoted distal regeneration.

**Conclusions:**

The chaperone system genes *hsp70*, *dnaJb4*, and *bag3* are significantly upregulated in the branchial sac vasculature following distal injury, defining a stress response that is essential for regeneration. The stress response is absent from distal fragments, but can be induced by a heat shock, which activates cell division in the branchial sac vasculature and promotes distal regeneration. This study demonstrates the importance of a stress response for stem cell activation and regeneration in a basal chordate, which may have implications for understanding the limited regenerative activities in other animals, including vertebrates.

**Supplementary Information:**

The online version contains supplementary material available at 10.1186/s12915-023-01633-y.

## Background

The program of regeneration includes inflammation, wound repair, cell proliferation, and the replacement of missing structures [1]. In vertebrates with limited regeneration capacities, such as mammals, wound repair typically terminates in fibrotic scar formation and is not followed by regeneration. In animals with higher regeneration capacities, wound repair and regeneration are linked, but the molecular relationship between these two processes is unclear.

The role of adult stem cells in regeneration has been studied in only a few model animals, highlighted by planarians [2], salamanders [3], and teleosts [4]. We have expanded these studies to include a non-traditional model, the tunicate *Ciona intestinalis* [5–7]. Tunicates have a long history of use as models for understanding the general principles of embryonic development [8] and are considered to be the chordate sister group of the vertebrates [9, 10]. Thus, tunicates offer a comparative approach to understanding stem cell contributions to regeneration as well as the evolution of regenerative activity within the Phylum Chordata [11, 12].

Tunicate embryogenesis is highly determinative: deletion of blastomeres or larval parts does not result in their replacement [13]. In contrast, development becomes more regulative following metamorphosis, when injuries to juveniles or adults can trigger the complete regeneration of missing tissues and organs [5, 14, 15]. In the colonial tunicates *Botryllus* and *Botrylloides*, zooids are accurately reproduced from vascular stem cells by whole body regeneration [11, 16, 17]. In the solitary tunicate *Polycarpa mytiligera*, complete regeneration is possible from three separated body fragments [18]. The solitary tunicate *Ciona* exhibits more-limited regeneration, which is known as unidirectional distal regeneration [5]. *Ciona* has an elongate cylindrical body with a distal region containing the oral siphon (OS), the neural complex (including the brain), and the atrial siphon, a middle sector containing a massive pharynx, highlighted by the branchial sac (BS), and a proximal region containing the heart, gonads, and digestive tract [19]. The OS is a feeding organ containing putative sensory organs (oral siphon pigment organs; OPO), interspersed between lobes of distal tissue, and bands of longitudinal and circular muscle (circular muscle bands, CMB), all of which are arranged in a distinct cylindrical pattern [15]. The underlying BS contains clusters of adult stem cells within its transverse vasculature, which are stimulated to proliferate by distal amputations or wounding and dispatch progenitor cells to the sites of injury [5, 6]. The progenitor cells migrate through sinuses of the open circulatory system [19]. When *Ciona* is bisected perpendicular to the proximal–distal axis, the proximal fragments regenerate distal tissues, such as the siphons and neural complex, but the distal fragments do not regenerate proximal viscera or completely replace distal structures, such as the OPO, CMB, and siphon lobes (after OS amputation), even when they contain part of the severed BS with accompanying stem cells [5, 7, 20]. The basis for this asymmetric regeneration is unknown, but because of the phylogenetic position of the tunicates within the chordates, it may be relevant to understanding limitations in the regenerative capacity of vertebrates.

OS regeneration in proximal body fragments begins with wound repair, apoptosis, and Wnt signaling at the injury site during the first day post-amputation [7, 15]. During the next 2–3 days, adult stem cells proliferate in the BS vasculature and progenitor cells are produced [5]. By 3 days post-amputation, the first progenitor cells have migrated distally and OS regeneration can be detected morphologically, a process that continues for 15–20 days until a complete OS is reformed [5, 6, 15]. Microarray and RNA sequencing analysis have shown that the Notch, TGFß, and Wnt signaling systems, a microRNA network; and the expression of conserved genes responsible for apoptosis and tissue repair are involved in OS regeneration [7, 21, 22]. The Notch signaling system is required for stem cell proliferation in the BS during regeneration [21]. In addition, roles for epigenetic modifiers and BMP1 in tissue repair have been reported during regeneration in *Botrylloides* [23] and *Polycarpa* [24], respectively. However, the molecular changes in the BS during *Ciona* regeneration and the underlying basis for differences in regenerative capacity between the proximal and distal body fragments are poorly understood.

Here we address the molecular basis of unidirectional regeneration in *Ciona* using new information generated from a BS transcriptome. The top upregulated genes in the transcriptome profile include the *hsp70* chaperone gene and the *dnaJb4* and *bag3* chaperone-related genes, which are expressed in BS vasculature cells previously identified as stem and progenitor cells [5]. siRNA-mediated gene knockdown showed that *hsp70* and *dnaJb4*, but not *bag3*, are required for progenitor cell accumulation at the injury site during OS regeneration. We show that complete OS regeneration, including reformation of the CMB, OPO, and siphon lobes, can be induced in distal body fragments containing part of the BS by a heat shock, suggesting that distal regeneration is dependent on the activation of a stress response.

## Results

### Differential gene expression in the branchial sac during oral siphon regeneration

**Fig. 1:**
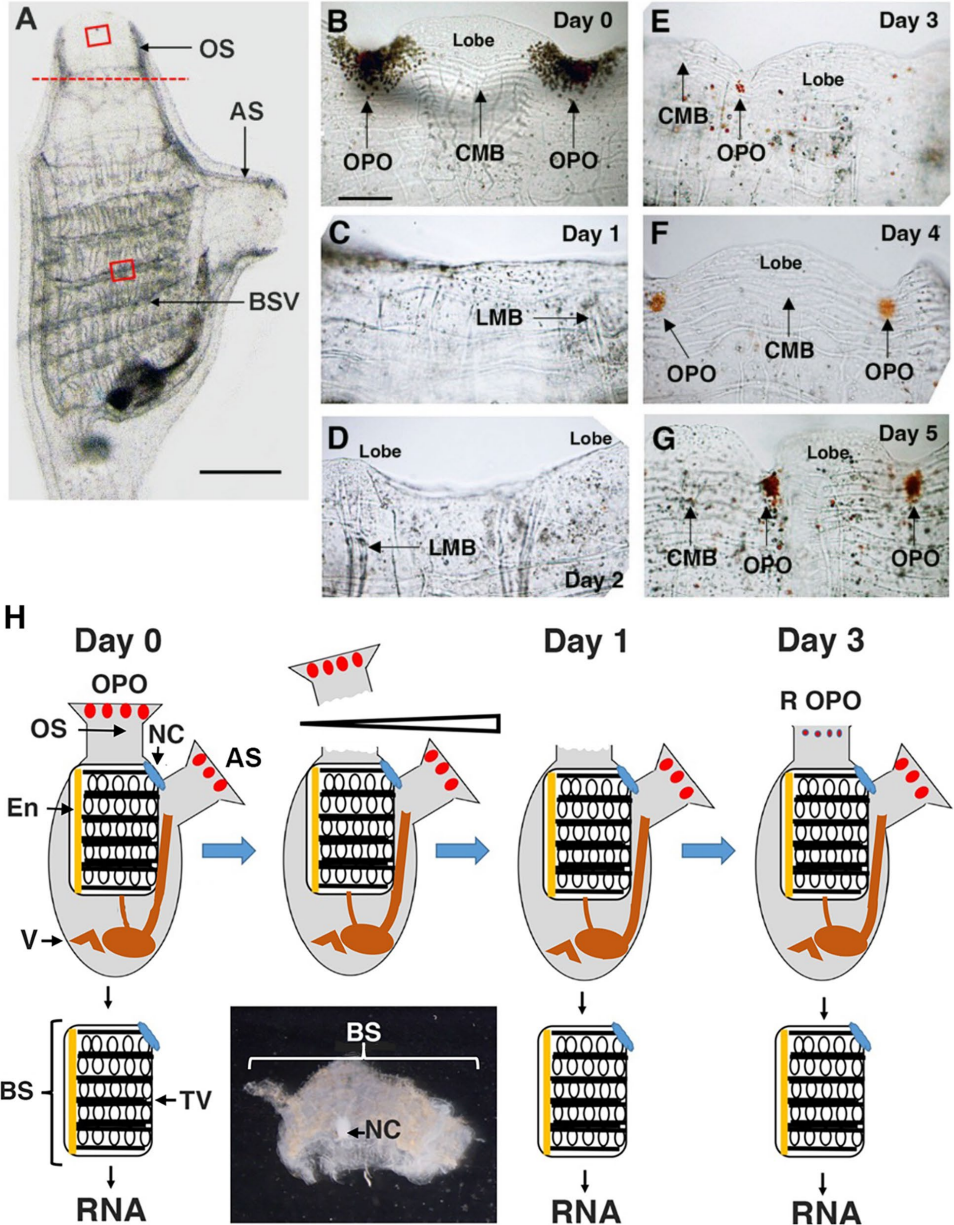
Progress of early regeneration after oral siphon amputation and branchial sac transcriptome experimental design. **A**–**G** The early steps of oral siphon (OS) regeneration. **A** A month-old unoperated animal showing the OS amputation plane (red dashed line). AS, atrial siphon; BSV, branchial sac vasculature. Red boxes: OS and BSV areas used for quantifying EdU labeling in Figs. 9 and 10. Scale bar: 50 μm. **B**–**G** The distal edges of the OS at day 0 in an unoperated animal (**B**) and at days 1–5 after OS amputation (**C–G**). OPO, oral siphon pigment organ; CMB, circular muscle band; LMB, longitudinal muscle band. Scale bar: 15 μm; same magnification in **B**–**G**. **H** Diagram illustrating BS isolation and RNA preparation design in day 0 unoperated controls and days 1 and 3 after OS amputation. Micrograph: An isolated BS. OPO, original oral siphon pigment organs; R OPO, regenerating OPO; NC, neural complex; AS, atrial siphon; En, endostyle; V, viscera; BS, branchial sac; TV, transverse vasculature with stem cells

We addressed the molecular basis of distal regeneration by sequencing RNA from isolated BSs after OS amputation. The early morphological changes that occur at the site of injury after OS amputation are shown in Fig. 1A–G. In unoperated (day 0) animals, the distal edge of the OS is highlighted by OPO, CMB, and siphon lobes (Fig. 1A, B). These structures are removed by OS amputation, and following a 2–3-day lag period the distal structures begin to be replaced during regeneration ([15]; Fig. 1C–E). At day 1 post-amputation, the OPO, CMB, and lobes are missing from the distal edge of the OS (Fig. 1C). The siphon lobes start to reform at day 2 (Fig. 1D), and the CMB and undifferentiated OPO first appear by day 3 (Fig. 1E). At days 4 and 5, the OPO begin to differentiate, more CMB are added, and the siphon lobes are further extended (Fig. 1F, G). Later stages of OS regeneration, which is completed in about 15–20 days, have been described previously [15].

Using the information described above as a guideline, we investigated differential gene expression in isolated BSs at day 1, during the gap between OS amputation and the beginning of regeneration, at day 3, during early OS regeneration, and at day 0, in controls with normal OSs. Three biological replicates of BSs were isolated and sequenced from each stage as illustrated in Fig. 1H.

**Fig. 2:**
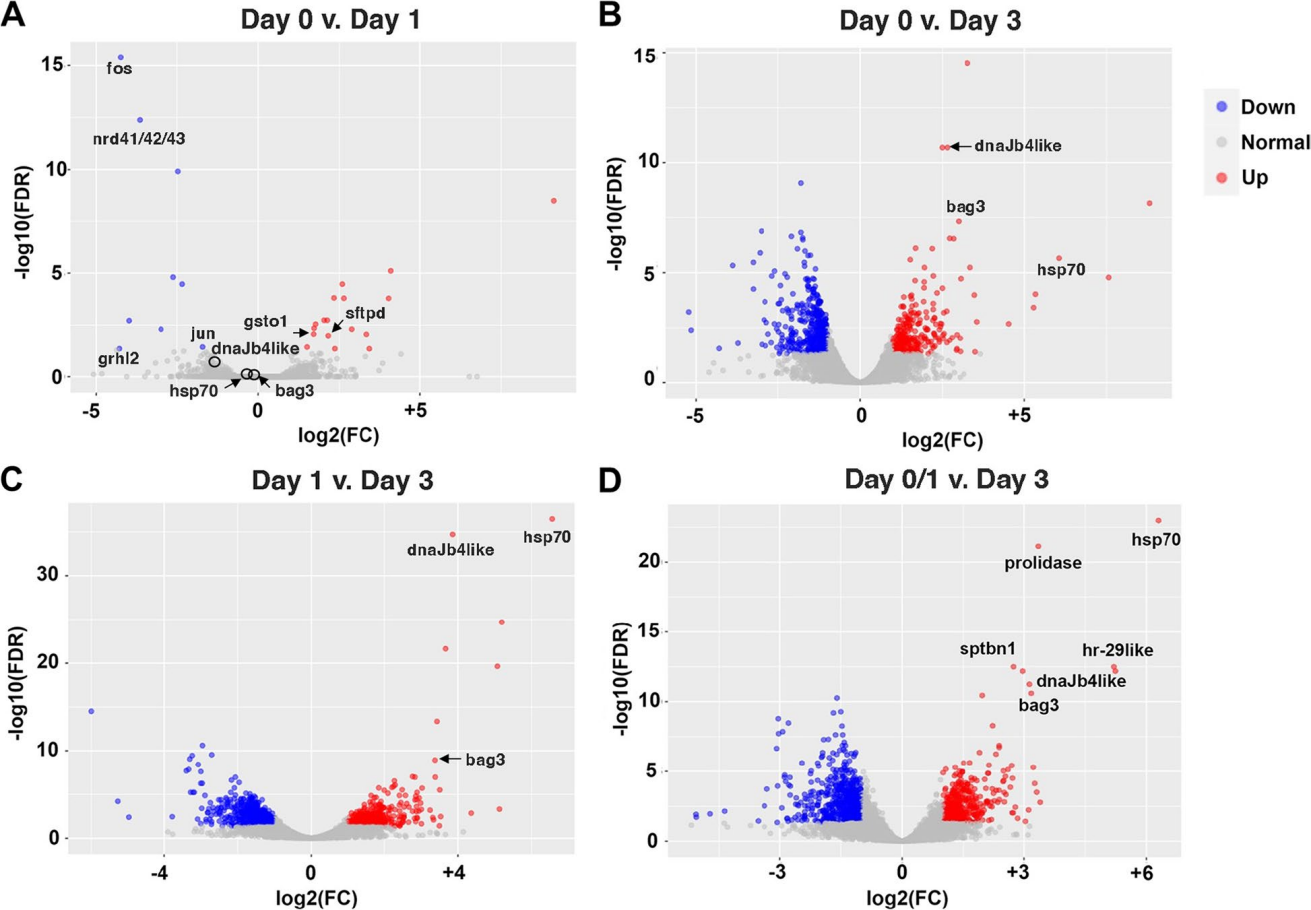
Volcano plots comparing differentially expressed genes between the day 0, day 1, and day 3 branchial sac samples. Gray dots: genes that are not significantly changed. Blue dots: significantly downregulated genes. Red dots: significantly upregulated genes. FC, fold change; FDR, false discovery rate

RNA sequencing generated 26,325,012 clean reads representing a total of 15,637 genes. The reads were mostly similar between the day 0 and day 1 BS samples, with the exception of a relatively small number of upregulated or downregulated transcripts (Fig. 2A; Additional file 1: Fig. S1; Additional file 2: Table S1.1, Table S1.2). The differentially expressed genes (DEGs) in the day 1 BS samples were highlighted by upregulated *gsto1*, encoding the glutamine-S-transferase omega enzyme involved in antioxidant metabolism [25]; upregulated *sftpd-like*, encoding the surfactant protein D involved in the innate immune response [26]; and downregulated *fos*, *jun*, *nrd41/42/43*, and *grhl2* encoding transcription factors [27–29] (Additional file 2: Table S1.1, Table S1.2). The downregulation of transcription factor genes suggests a change in gene regulation in the BS following OS amputation. Genes involved in immediate tissue repair were not detected in the day 1 profile, perhaps because OS amputations did not wound the BS (Fig. 1H).

The reads of the day 3 BS samples differed from the day 0 and day 1 BS samples (Fig. 2B, C; Additional file 2: Fig. S1). Comparison of the day 3 BS samples to the combined day 0 and day 1 (day 0/1) samples identified 1149 DEG consisting of 517 upregulated genes and 632 downregulated genes (Fig. 2D; Additional file 3: Table S2.1, Table S2.2). The molecular chaperone-encoding gene *hsp70* was the most highly upregulated gene in the profile, and the HSP70 co-chaperone *dnaJb4-like* (*dnaJb4*) and the HSP70 chaperone-regulating *bag3-like* (*bag3*) genes were also highly upregulated in the day 3 versus day 0/1 BS samples (Fig. 2D). The *hr-29* gene, encoding an ascidian muscle protein in the stress-related, small heat shock protein/alpha crystallin family [30, 31]; the *prolidase* gene, encoding a metalloprotease involved in inflammation, wound healing, and cell proliferation [32]; and the *sptbn1* gene, encoding a ß2-spectrin, which functions in cell adhesion, cell spreading, apoptosis, and cell cycle regulation [33], were other highly upregulated genes in the transcriptome profile (Fig. 2D; Additional file 3: Table 2.1). Most of the downregulated genes in the day 3 samples were related to metabolic and homeostatic processes (Fig. 2; Additional file 3: Table S2.2), consistent with the role of BS stem cells in pharyngeal cell recycling during homeostasis [7]. The results suggest that by day 3 following OS amputation, BS gene expression profiles shift from transcripts associated with metabolism and homeostasis to those related to tissue repair and regenerative processes.

### Weighted gene correlation network analysis

**Fig. 3:**
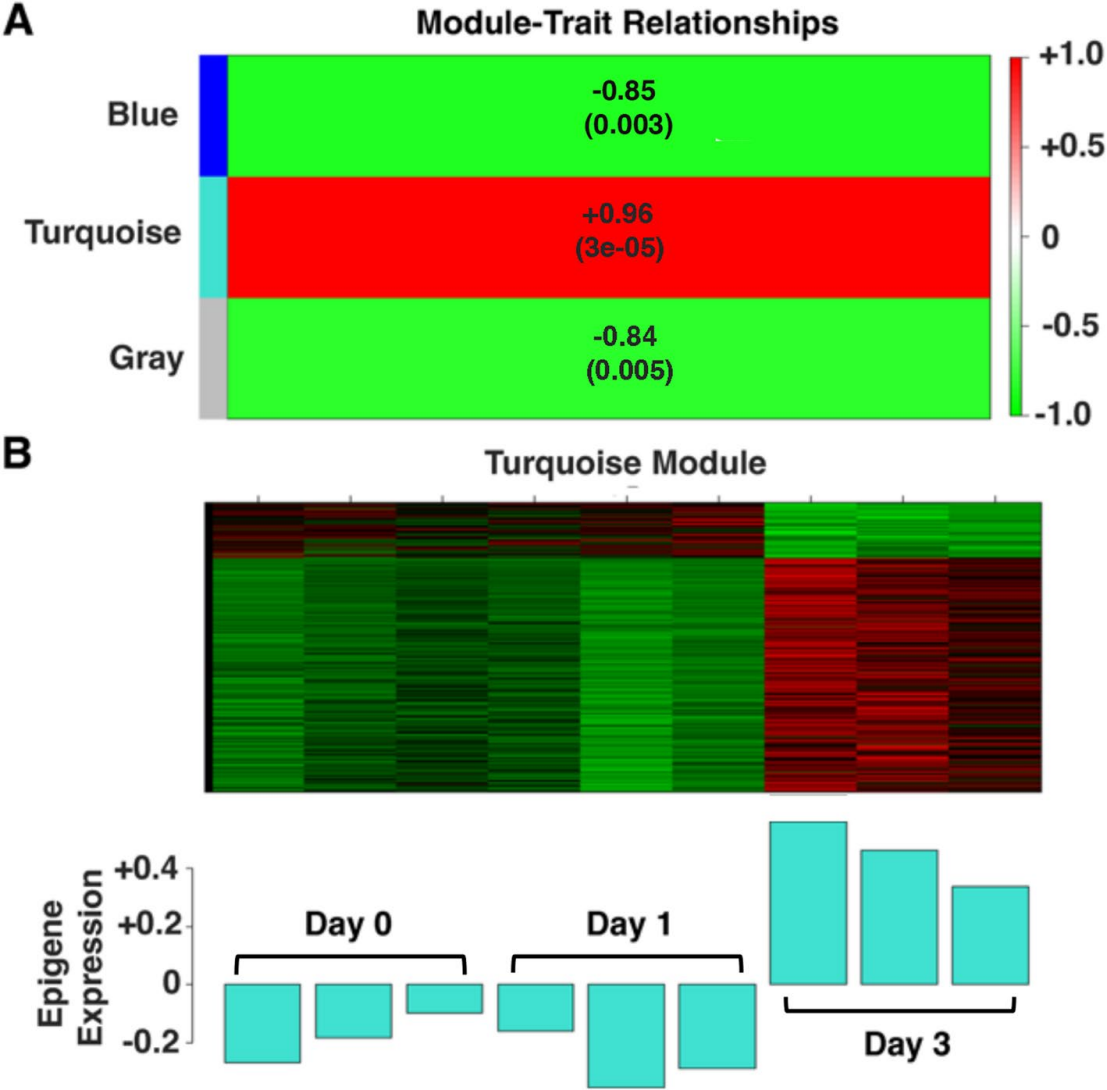
Organization of differentially expressed branchial sac genes into modules by weighted gene correlation network analysis. **A** Correlation of blue, turquoise, and gray modules with regeneration in day 3 branchial sac (BS) samples. The *p* values show significance of correlation in each module. **B** Top: Heat map of differentially regulated genes in the turquoise module in 3 replicates of day 0, day 1, and day 3 BS samples. Bottom: Epigene expression in day 0, day 1, and day 3 BS replicates

Weighted gene correlation network analysis (WGCNA) was performed to identify genes correlated with regeneration in the day 3 BS samples. The 1149 DEG were organized into three modules by WGCNA. The blue module, which was negatively correlated with regeneration, was composed entirely of 423 downregulated DEG (Fig. 3A; Additional file 4: Table S3.1). The turquoise module, which was positively correlated with regeneration (*P* < 0.0001, *r*^2^ > 0.95; Fig. 3A, B; Additional file 4: Table S3.2), was composed of 640 DEG consisting of 517 upregulated and 123 downregulated genes. The most highly upregulated gene in the turquoise module was *hsp70*. The gray module was composed of the remaining 86 DEG (Additional file 4: Table S3.3), which did not group into a single module and thus was not analyzed further.

**Fig. 4:**
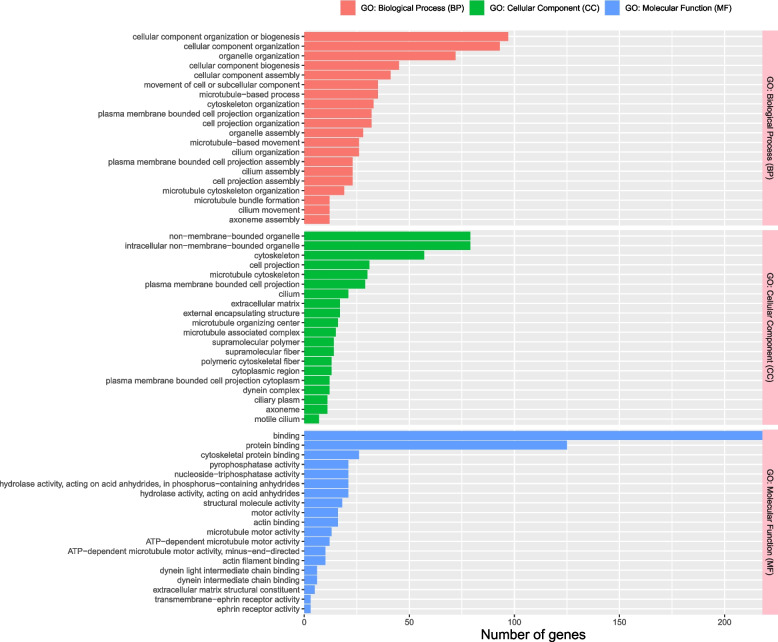
Gene ontology (GO) enrichment analysis of turquoise module genes. The number of turquoise genes in the top 20 GO terms for Biological Process, Cellular Component, and Molecular Function is shown

Pathway enrichment analysis was done to predict the function of genes in the blue and turquoise modules. GO analysis showed that the blue module genes are enriched in biological processes, including organic substance biosynthetic process, cellular biosynthetic process, cellular protein metabolic process, peptide metabolic process, ATP metabolic process, electron transport chain, and oxidative phosphorylation, which suggest roles in metabolism and homeostasis (Additional file 5: Fig. S2). In contrast, the turquoise module genes are enriched in potential functions such as binding, protein binding, cellular component organization or biogenesis, intracellular non-membrane bound organelle, organelle organization, and cytoskeleton (Fig. 4), which suggest tissue repair and regenerative functions.

**Fig. 5:**
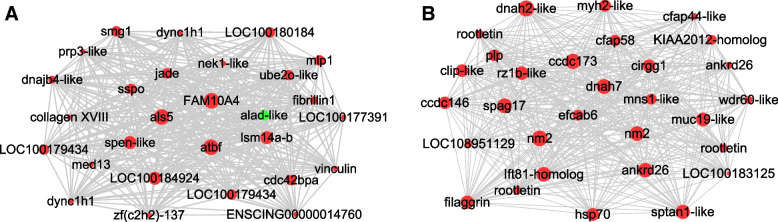
Hub gene co-expression networks in the turquoise module. **A** A network showing the top 28 hub genes with the highest degrees of connectivity identified by Cytoscape. **B** A network showing the top 30 hub genes with lower degrees of connectivity identified by the Density of Maximum Neighborhood Component method in Cytoscape CytoHubba. Green spheres: downregulated genes. Red spheres: upregulated genes. The area of the spheres represents the degree of correlation of each hub gene in the networks

The hub genes in the turquoise module were identified by the degree of connectivity (Fig. 5; Additional file 6: Table S4.1). Some of the notable hub genes of highest connectivity included the molecular chaperone *dnaJb4*; *atbf1*, encoding a transcription factor regulating the cell cycle and neurogenesis [34]; *nek1*, encoding a cell cycle regulating serine threonine kinase [35]; *dync1.1*, encoding dynein heavy chain 1 [36]; *vinculin*, encoding an actin filament binding protein [37]; and *prp3-like*, encoding a predicted RNA binding protein in the spliceosome [38]. The *hsp70* gene was identified as a hub gene of lower connectivity (Fig. 5B; Additional file 6: Table S4.2). The turquoise module hub genes provide a resource for further studies of the molecular basis of *Ciona* regeneration.

### HSP70 system chaperone complex

**Fig. 6:**
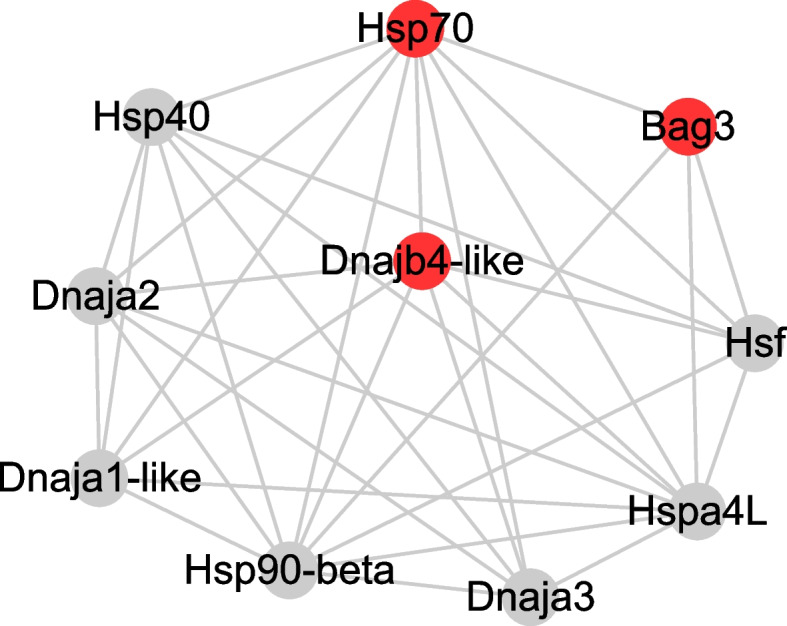
STRING predicted protein interaction network with HSP70. Red spheres: upregulated genes. Gray spheres: genes with unchanged expression levels. DNAJb4-like and BAG3 showed interaction scores with HSP70 of 0.975 and 0.951, respectively

STRING analysis was conducted to predict protein–protein interactions with HSP70. The results suggested direct interactions of DNAJb4 and BAG3 with HSP70 (Fig. 6; Additional file 7: Table S5), implying that these genes may function in an HSP70 chaperone system during *Ciona* regeneration. Accordingly, the *hsp70*, *dnaJb4*, and *bag3* genes were focused on during the remainder of this investigation.

### HSP70 system genes are upregulated in branchial sac vasculature cells during oral siphon regeneration

**Fig. 7:**
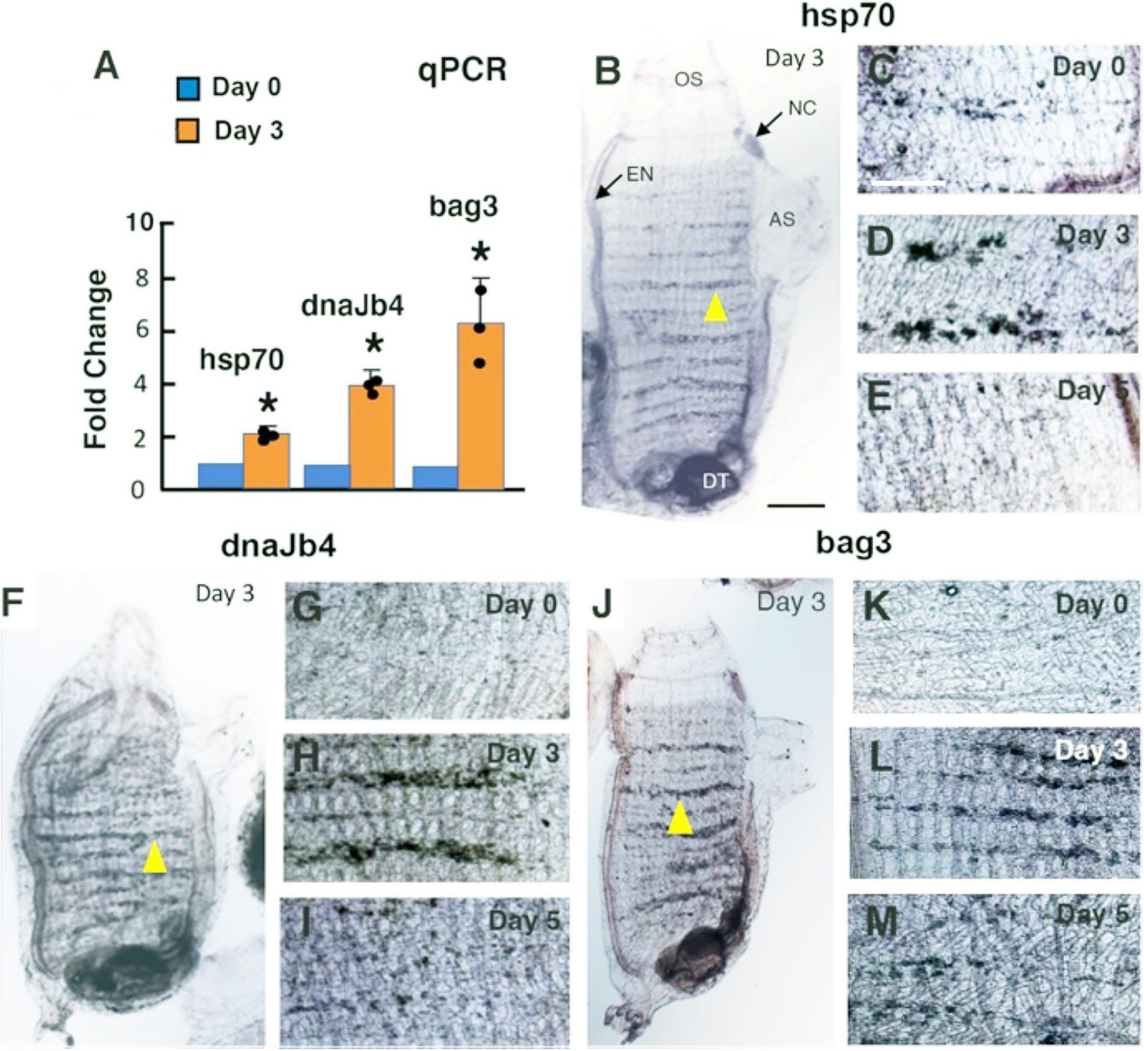
Chaperone gene expression during oral siphon regeneration. **A** qRT-PCR analysis of *hsp70*, *dnaJb4*, and *bag3* transcript levels at day 3 after oral siphon (OS) amputation compared to day 0 controls. Asterisks: significance (*p* = .000087, *p* < .00001, and *p* = .001156 from left to right) by one-tailed Student’s *t* test with ∆Ct. **B**–**M** *hsp70* (**B**–**E**), *dnaJb4* (**F**–**I**), and *bag3* (**J**–**M**) unoperated controls at day 0 (**C**, **G**, **K**) and regenerating animals at day 3 (**B**, **D**, **F**, **H**, **J**, **L**), and day 5 (**E**, **I**, **M**) after OS amputation. **B**, **F**, **J** Entire animals. **C**–**E**, **G**–**I**, and **K**–**M** Magnified regions of the branchial vasculature (arrowheads in **B**, **F**, **I**). The digestive tract (DT) is unstained but appears dark due to the presence of food and feces. NC, neural complex; AS, atrial siphon; EN, endostyle. Scale bar in **B**: 50 μm; same magnification in **B**, **F**, **J**. Scale bar in **C**: 15 μm; same magnification in **C**–**E**, **G**–**I**, and **K**–**M**. Data from three replicate experiments

Upregulation of the *hsp70*, *dnaJb4*, and *bag3* genes and their expression pattern during OS regeneration were determined by qRT-PCR and in situ hybridization, respectively. Quantification showed that *hsp70*, *dnaJb4*, and *bag3* mRNA levels increased at Day 3 post-amputation relative to Day 0 controls (Fig. 7A), coinciding with the appearance of undifferentiated OPO and the first CMB during early OS regeneration (Fig. 1E). To determine the location of chaperone gene expression in the BS and other parts of the body, we performed in situ hybridization of whole animals before and after OS amputation (Fig. 7B–M). The results showed that all three genes were expressed at low levels in BS vasculature cells of day 0 controls (Fig. 7C, G, K). The expression of *hsp70*, *dnaJb4*, and *bag3* expression was stronger at day 3 (Fig. 7B, D, F, H, J, L), corresponding to the beginning of OS regeneration (Fig. 1A–G), and then declined at day 5 (Fig. 7E, I, M). Chaperone gene expression was detected in BS vasculature cells, but not in the regenerating OS (Fig. 7B, F, J). Expression of the chaperone genes was also detected in the neural complex and endostyle in OS-amputated animals and controls (Fig. 7B, F, J). The results indicate that *hsp70*, *dnaJb4*, and *bag3* are upregulated in BS vasculature cells previously identified as stem and progenitor cells [5, 6] during OS regeneration.

### The *hsp70* and *dnaJb4* genes are required for oral siphon regeneration

**Fig. 8:**
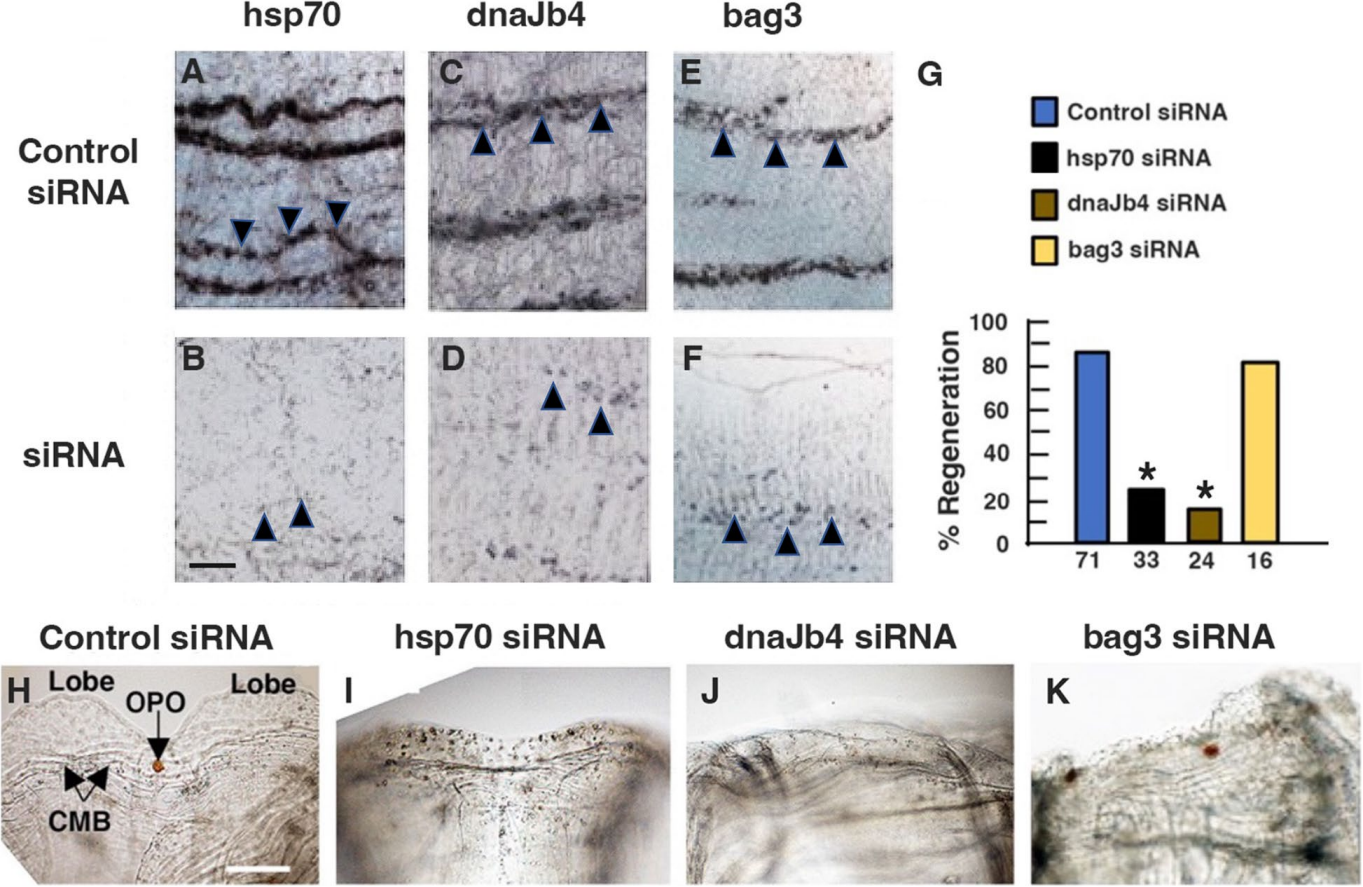
Effects of siRNA knockdown on chaperone gene expression and oral siphon regeneration. **A**–**F** In situ hybridization showing *hsp70* (**A**, **B**), *dnaJb4* (**C**, **D**), and *bag3* (**E**, **F**) expression in the branchial sac vasculature (arrowheads) in control siRNA (**A**–**E**), *hsp70* siRNA (**B**), *dnaJb4* siRNA (**D**), and *bag3* siRNA (**F**) treated animals at day 5 after OS amputation. Scale bar: 20 μM; same magnification in **A**–**F**. **G**–**K** Effects of siRNA gene knockdown on oral siphon (OS) regeneration. **G** Percent regeneration in control, *hsp70*, *dnaJb4*, and *bag3* siRNA-treated animals at day 5 after OS amputation. The number of animals is shown at the base of each bar. Asterisk: significance (*χ*^2^ = 53.23,* p* < .000001) by chi-square test. **H**–**K** Distal edges of the OS at day 5 after OS amputation in control siRNA (**H**), *hsp70* siRNA (**I**), *dnaJb4* siRNA (**J**), and *bag3* (**K**) siRNA-treated animals. OPO, oral siphon pigment organ; CMB, circular muscle bands. Combined data from 5 replicate experiments

Short interfering RNA (siRNA) gene knockdown [7] was used to study the relationship between chaperone gene expression and OS regeneration. After OS amputation, proximal fragments were incubated with *hsp70* siRNA, *dnaJb4* siRNA, *bag3* siRNA, or control siRNA; at day 2, they were washed into fresh siRNAs; incubation with siRNAs was continued until day 5; and the effects on regeneration were determined. We used in situ hybridization to determine the effects of the siRNAs on chaperone gene expression specifically in the BS vasculature, and the results showed that the chaperone gene siRNAs, but not the control siRNA, were effective in knocking down *hsp70*, *dnaJb4*, and *bag3* expression (Fig. 8A–F). Knockdown of the *hsp70* or *dnaJb4* genes prevented CMB, OPO, and siphon lobe regeneration (Fig. 8G–J), but control siRNA had no effects on OS regeneration (Fig. 8G, H). In contrast, although *bag3* siRNA was effective in knocking down *bag3* gene expression in the BS vasculature (Fig. 8E, F), replacement of the OPO, CMB, or siphon lobes was not prevented (Fig. 8G, K), suggesting that the *bag3* gene is not required for regeneration. These results show that *hsp70* and *dnaJb4* expressions are necessary for OS regeneration.

### Knockdown of *hsp70* and *dnaJb4* affects progenitor cell accumulation in the regenerating oral siphon

**Fig. 9:**
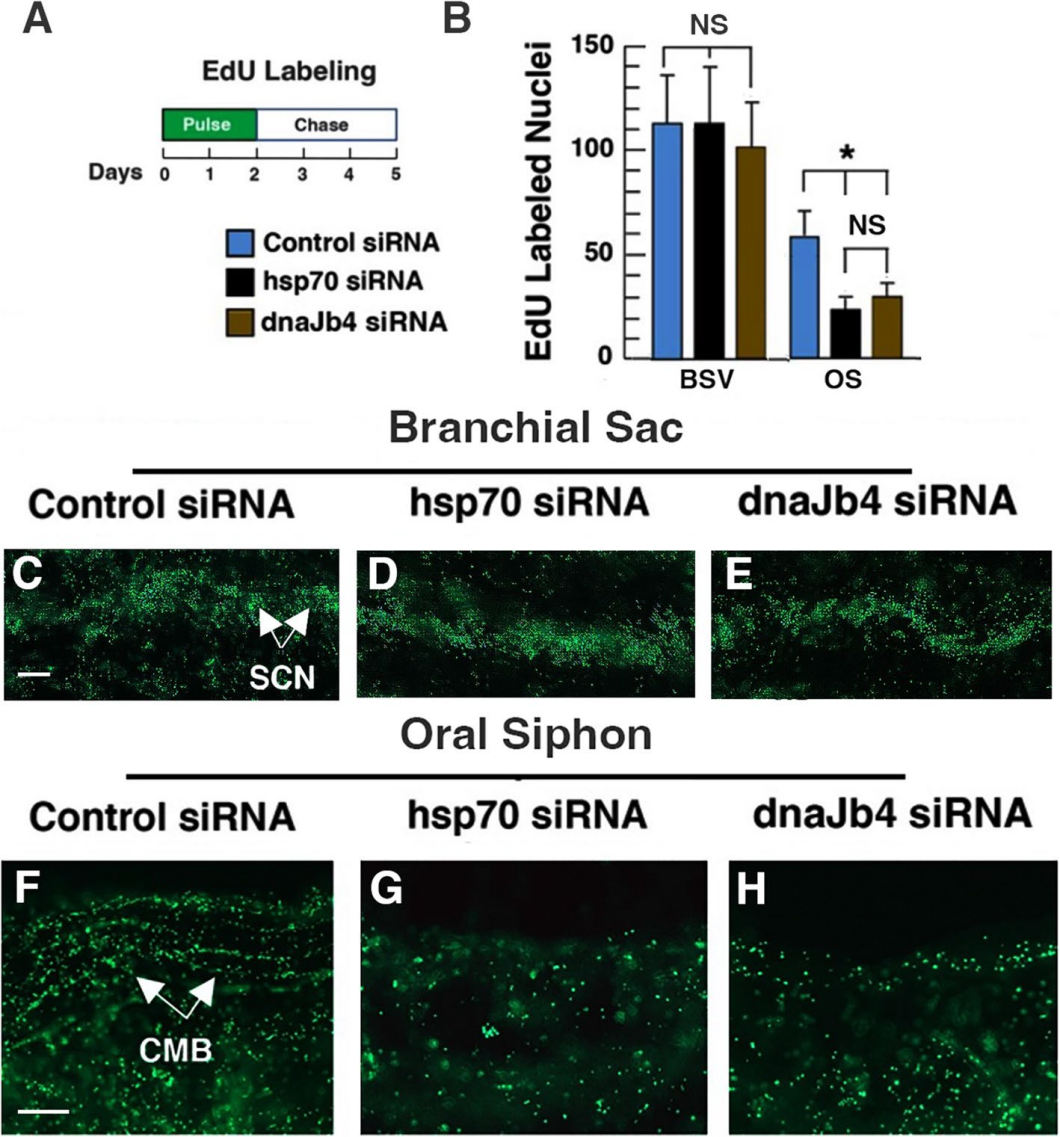
Effects of siRNA knockdown on EdU labeling in the branchial sac and the amputated oral siphon. **A** The EdU pulse-chase labeling regime used in these experiments. OS amputation occurred at day 0 and was followed immediately by siRNA and EdU incubation, and EdU was chased and fresh siRNA added at day 2. **B**–**H** EdU labeling in the branchial sac vasculature (**B**–**E**) and oral siphon (OS) (**B**, **F**–**H**) in control siRNA (**C**, **F**), *hsp70* siRNA (**D**, **G**), and *dnaJb4* siRNA (**E**, **H**) labeled animals at day 5 after OS amputation and EdU pulse-chase labeling. CMB, circular muscle bands; SCN, branchial sac vasculature. Scale bar: 10 μm: same magnification in **C**–**E** and **F**–**H**. See Fig. 1A for location of EdU quantification areas. EdU labeling of 10 animals was determined for each bar. NS, no significance. Asterisk: significance (*f* = 52.32, df = 27, *p* < .00001) by one-way ANOVA with post hoc Tukey HSD. Combined data from three replicate experiments

EdU pulse-chase experiments conducted previously have shown that cell proliferation begins in the BS vasculature within the first day after OS amputation, and labeled progenitor cells migrate distally and accumulate in the CMB of the regenerating OS beginning about 2–3 days after OS amputation [5]. Similar experiments using a 2-day EdU pulse and 3-day chase (Fig. 9A) were carried out to determine the effects of *hsp70* and *dnaJb4* gene knockdown on the proliferation of BS vasculature cells and progenitor cell targeting. Since *bag3* was not required for OS regeneration (Fig. 8G, K), it was excluded from this study. The OS was amputated and animals were treated with *hsp70* siRNA, *dnaJb4* siRNA, or control siRNA according to the schedule described above; the EdU pulse-chase regime was initiated immediately after OS amputation; and EdU labeling was compared in the BS vasculature and the OS in amputated and control animals at the end of the chase (Fig. 9A). There were no significant differences in EdU labeling in the BS vasculature cells of *hsp70* or *dnaJb4* siRNA-treated animals compared to controls (Fig. 9B–E), suggesting that the expression of these genes is not required for cell proliferation. In contrast, EdU labeling was significantly reduced in the regenerating OS of *hsp70* siRNA or *dnaJb4* siRNA-treated animals compared to controls (Fig. 9B, F–H). These results are consistent with a role for the *hsp70* and *dnaJb4* genes in progenitor cell accumulation in the regenerating OS.

### Heat shock induction of stress and rescue of regenerative activity in distal fragments

**Fig. 10:**
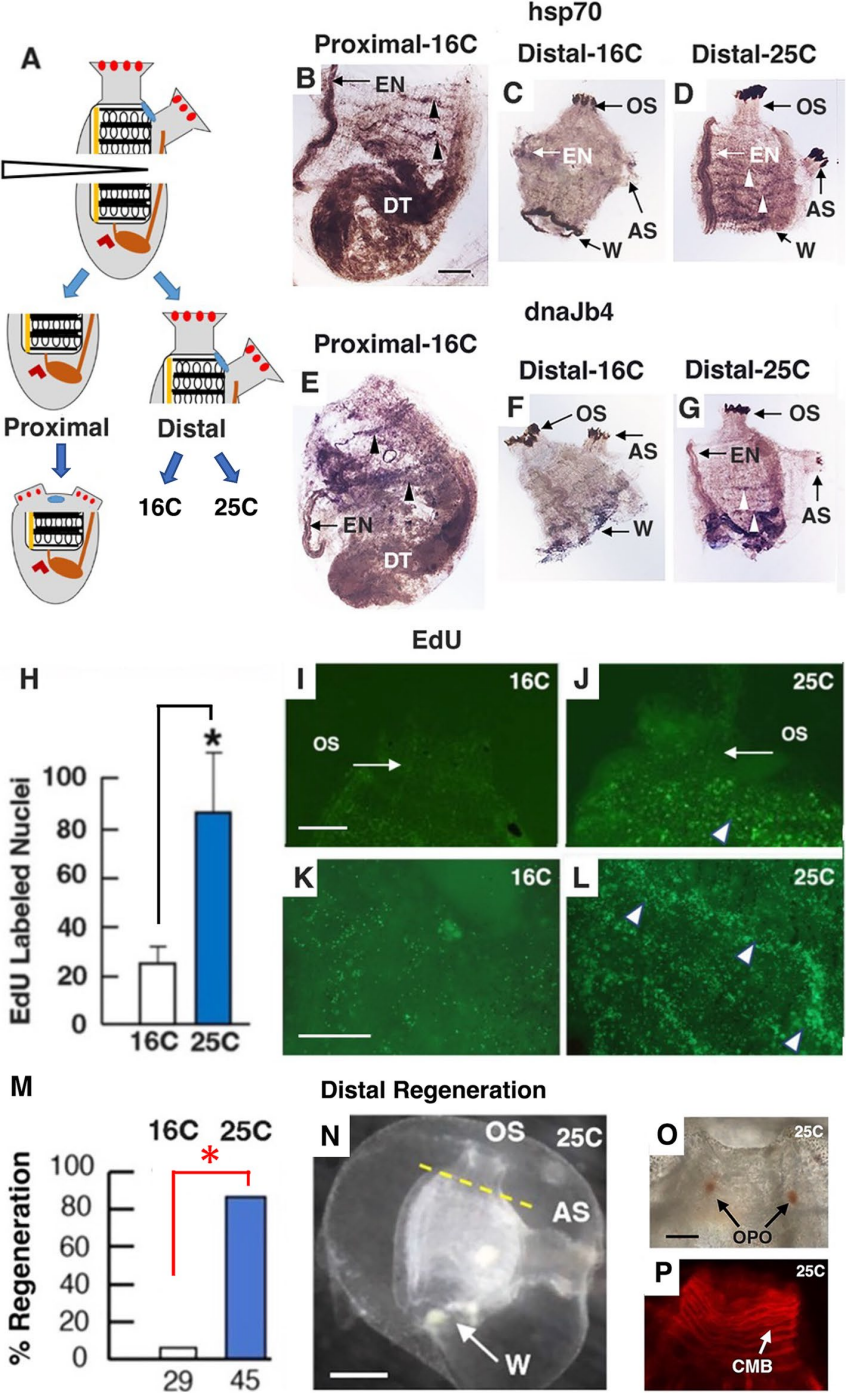
Effect of a heat shock on *hsp70* and *dnaJb4* expression, cell proliferation, and regeneration of distal fragments. **A** The experimental design for producing distal and proximal fragments by bisection. The proximal fragment regenerates distal structures, but the distal fragment does not regenerate proximal structures. Distal fragments are maintained at 16 °C or subjected to 25 °C. See Fig. 1H for labels. **B**–**G** In situ hybridization showing *hsp70* or *dnaJb4* expression in the branchial sac vasculature (arrowheads) of distal (**C**, **F**) or proximal (**B**, **E**) fragments cultured at 16 °C and 25 °C heat-shocked distal fragments (**D**, **G**). EN, endostyle. Arrows indicate pigmentation in the oral siphon (OS), atrial siphon (AS), and bisection plane (W). DT, digestive tract. Scale bar: 30 μm; same magnification in **B**–**G**. **H**–**L** EdU labeling. **H** Quantification of EdU labeling in the branchial sac vasculature of heat-shocked and control distal fragments. EdU quantification areas are shown in Fig. 1A. Asterisk: significance (*t* =  − 5.67, df = 18, *p* < .00001) by Student’s one-tailed *T* test. **I**–**L** EdU labeling in the OS (**I**, **J**) and branchial sac vasculature (arrowheads) (**K**, **L**) of a control (**I**,** K**) and a heat-shocked (**J**, **L**) distal fragment. Scale bars: 10 μm; same magnification in **I, J** and **K**, **L**. **E**, **M**–**P** OS regeneration in heat-shocked distal fragments. **M** Percentage of OS regeneration in heat-shocked and control distal fragments. The number of distal fragments assayed is shown at the base of each bar. Asterisk: significance (*χ*^2^ = 46.4129, *p* < .000001) by a chi-square test. **N** A heat-shocked distal fragment showing OS regeneration at 8 days post-amputation. Dashed line: original amputation plane. AS, atrial siphon; W, proximal wound site. Scale bar: 50 μm. **O**–**P** OS regeneration in heat-shocked distal fragments at 8 days post-amputation showing **O** oral siphon pigment organs (OPO) and **P** phalloidin-labeled circular muscle bands (CMB). Scale bar: 10 μm; same magnification in **O**, **P**. Combined data from three or more replicate experiments

When *Ciona* is bisected perpendicular to the proximal–distal axis, distal regeneration, including OS replacement, occurs in proximal fragments but is absent or incomplete in distal fragments, even though the latter contain part of the BS with stem cells (Fig. 10A) [5, 7]. To determine whether proximal and distal fragments activate a stress response, *hsp70* and *dnaJb4* expression was investigated by in situ hybridization after bisection (Fig. 10B, C, E, F). The results showed that *hsp70* and *dnaJb4* were expressed in the BS vasculature of the proximal fragments (Fig. 10B, E) but gene expression was not detected in the BS vasculature of distal fragments (Fig. 10C, F). These results opened the possibility that limited regeneration in distal fragments could be explained by the lack of a stress response. To test this hypothesis, a stress response was induced in the distal fragments by applying a heat shock. *Ciona* is raised in the laboratory at 16 °C, which was also the temperature at which surgical operations were done in the experiments described above. To select an appropriate stress regime, animals were bisected (Fig. 10A), distal fragments were exposed to temperatures between 18 and 28 °C for 30 min, and 25 °C was found to be the highest temperature with maximal survival. When animals were bisected and the distal fragments were assayed by in situ hybridization immediately after a 30 min 25 °C heat shock (Fig. 10A), they showed stronger *hsp70* and *dnaJb4* expression in the BS vasculature than controls maintained at 16 °C (Fig. 10C, D, F, G), suggesting that stress was successfully induced. Next, cell proliferation was compared by incubating the heat-shocked and control distal fragments with EdU for 2 days. The results showed that EdU labeling was significantly higher in the BS vasculature of the heat-shocked distal fragments compared to the controls (Fig. 10H, K, L), and no EdU labeling was seen in the OS of either the heat shocked or control distal fragments (Fig. 10I, J). Lastly, regeneration was compared in the heat-shocked and control distal fragments after OS amputation and culture at 16 °C for 8 days post-amputation (Fig. 10M–P). Most of the control distal fragments showed no OS regeneration or incomplete OS regeneration, involving replacement of 1–3 OPO [5] but no siphon lobes or CMB (Fig. 10M; Additional file 8: Table S6). In contrast, more than 80% of the heat-shocked distal fragments replaced a complete OS with OPO, 6-8 siphon lobes, and multiple rows of phalloidin-stained CMB (Fig. 10M–P; Additional file 8: Table S6). As described previously [7], the proximal fragments regenerated a new OS, an atrial siphon, and a neural complex, but viscera were not replaced in either heat-shocked or control distal fragments (Fig. 10N), even after culture for up to 2 weeks, a result consistent with previous studies showing unidirectional body regeneration [5]. However, the proximal bisection plane became darkly pigmented (Fig. 10C, D, F, G), possibly a sign of wound repair [6].

In summary, the results show that cell proliferation in the BS vasculature and complete regeneration of the OS in distal fragments can be induced by a heat shock.

## Discussion

Distal body injuries in *Ciona* activate BS stem cells to produce progenitor cells that undergo long distance migration to replace CMB in the siphons, the neural complex, and probably other distal tissues [5, 6]. Here we describe an analysis of differential gene expression in the BS after OS amputation and use the information provided by the transcriptome profiles to obtain new insights into why *Ciona* distal body fragments are normally unable to undergo regeneration.

### Differential gene expression in the branchial sac during regeneration

Transcriptome analysis of the BS revealed 1149 genes, including 632 downregulated and 517 upregulated genes, during early OS regeneration. WGCNA separated the differentially regulated genes into two major hub modules, one containing downregulated genes, which are mostly related to metabolism and homeostasis, and another containing primarily upregulated genes related to regeneration. Most of the differentially regulated BS genes did not overlap with genes identified previously during OS regeneration [21, 22], implying that different molecular events may occur during distal injury repair at the wound site and stem cell activation in the BS. The divergence in predicted functions between the two major WGCNA modules suggests that distal injuries cause BS stem cells to switch from their normal roles in growth, including hematogenesis [39] and cyclic replacement of ciliated cells lining the BS fissures [7], to new functions in replacing the CMB and other siphon tissues and organs. It is notable that immediate tissue repair genes were not detected in the BS transcriptome at day 1 after OS amputation, as might be anticipated because the BS contains a major system of body vasculature and would be expected to contain circulating injury-related (including immune) cells. Accordingly, we speculate that the BS vasculature could be restricted to outgoing cells, such as hemocytes and progenitor cells produced within the stem cell niches [5, 39], and may exclude cells circulating inward from other parts of the body. The collection of differentially regulated BS genes obtained in this investigation will serve as a valuable resource for future molecular analysis of *Ciona* regeneration.

The transcriptome profiles show that some of the top upregulated genes in the BS encode chaperones and related proteins with predicted functions in a heat shock-like stress response. The highest upregulated gene in the transcriptome encodes HSP70, a key component of the cellular network of molecular chaperones responsible for proper protein folding, assembly, and other post-translational functions [40, 41]. The DNAJ family member and co-chaperone with HSP70, DNAJB4, and the chaperone-associated protein BAG3, which assists HSP70 in carrying out some of its functions [41], were also encoded by top upregulated genes in the BS transcriptome. Two of these genes, *hsp70* and *dnaJb4*, were identified as hub genes in the WGCNA turquoise module, consistent with important roles in regeneration. The presence of these DNAJB and BAG family members in the transcriptome profile, suggests that HSP70, DNAJB4, and BAG3 work together during *Ciona* distal regeneration. Supporting this conclusion, STRING analysis predicted direct interactions between HSP70 and the DNAJB4 and BAG3 proteins. This chaperone system may facilitate the function of proteins involved in the replacement of an injured OS and other distal organs. Therefore, future studies directed toward the identification of the client proteins chaperoned by the HSP70/DNAJB4/BAG3 system may reveal some of the key molecular regulators of regeneration.

### Upregulation of HSP70 system genes reveals a stress response required for regeneration

The upregulation and localization of *hsp70*, *dnaJb4*, and *bag3* expression in BS vasculature containing stem and progenitor cells during early regeneration were confirmed by qRT-PCR and in situ hybridization, respectively, and their critical roles in distal regeneration were supported by siRNA-mediated gene knockdown. The siRNA studies showed that the major tissues and organs replaced after OS amputation-the OPO, CMB, and siphon lobes could be suppressed by knockdown of the *hsp70* and *dnaJb4* genes, but not the *bag3* gene, suggesting that BAG3 is not required for regeneration. The results are consistent with demonstrations of the importance of similar heat shock genes in other regenerating systems, including planarian whole body regeneration [42, 43], starfish arm regeneration [44], zebrafish fin regeneration [45], *Xenopus* tadpole hindlimb regeneration [46], and mouse liver regeneration [47]. Our studies contribute a new understanding of the process of *Ciona* unidirectional regeneration by identifying stress in the BS as a key event necessary for initiating the distal regeneration program.

The relationship between BS stem cell function and the chaperone system was revealed by applying a EdU pulse chase to siRNA-treated regenerating animals. Cell proliferation was not affected in the BS, but the accumulation of progenitor cells in the regenerating OS was substantially decreased, suggesting possible effects on the dispatch, migration, proliferation, or survival of progenitor cells. A similar result was obtained when apoptosis, which occurs on the proximal side of distal wounds [6], was suppressed by caspase inhibitors [7]. The role of the HSP70 chaperone system in progenitor cell migration and not stem cell proliferation has also been reported in planaria [48], and HSP70 and the HSP40 family member MRJ are known to regulate cell migration in tumor cells [49]. In *Ciona*, our results imply that the activation of stem cell proliferation may be independent of the HSP70 chaperone system expressed in the BS. Thus, of the presently known molecular events at the wound site or in the BS, only Notch signaling appears to be connected to the activation of stem cell proliferation [21].

### Heat shock stress induces oral siphon regeneration in distal body fragments

A longstanding question in *Ciona* regeneration research has been why distal fragments, in contrast to proximal fragments, do not regenerate after body bisection [20, 50]. This asymmetric regeneration in *Ciona* contrasts strikingly to the regeneration of complete animals from all three fragments after body trisection in another solitary tunicate, *Polycarpa mytiligera* [18]. In *Ciona*, the BS is required [20], but not sufficient [7], for regeneration. Thus, it is clear that distal fragments lacking part of the BS, such as amputated OSs, do not regenerate because they are missing BS stem cells. However, the absence of regenerative activity in *Ciona* distal fragments containing a part of the BS cannot be attributed to the absence of stem cells, which have been documented in both severed parts of this organ [7]. In this investigation, we discovered that *hsp70* and *dnaJb4* expression occur normally in the BS of proximal but not distal fragments, suggesting that distal fragments fail to regenerate because a stress response defined by the HSP70 system is not activated, presumably due to the absence of a factor localized in the proximal fragments. To test this hypothesis, we applied a brief heat shock to distal fragments, which was successful in increasing *hsp70* and *dnaJb4* expression in BS vasculature cells, activating cell proliferation, and rescuing OS regeneration. There are reports that mammalian nerve and muscle regeneration and the survival of mesenchymal stem cells can be improved by increased temperature [51–53]. To our knowledge, however, our study is unique in showing that heat treatment can rescue regeneration of an entire animal body part. It is known that non-lethal heat treatment in *Ciona* increases the expression of 6 HSP70 superfamily genes, 8 DNAJ protein family genes, and 2 BAG family genes [54]. Therefore, it remains to be determined whether the HSP70 chaperone system genes described here, and/or other factors activated by heat stress, are responsible for the rescue of distal regeneration.

**Fig. 11:**
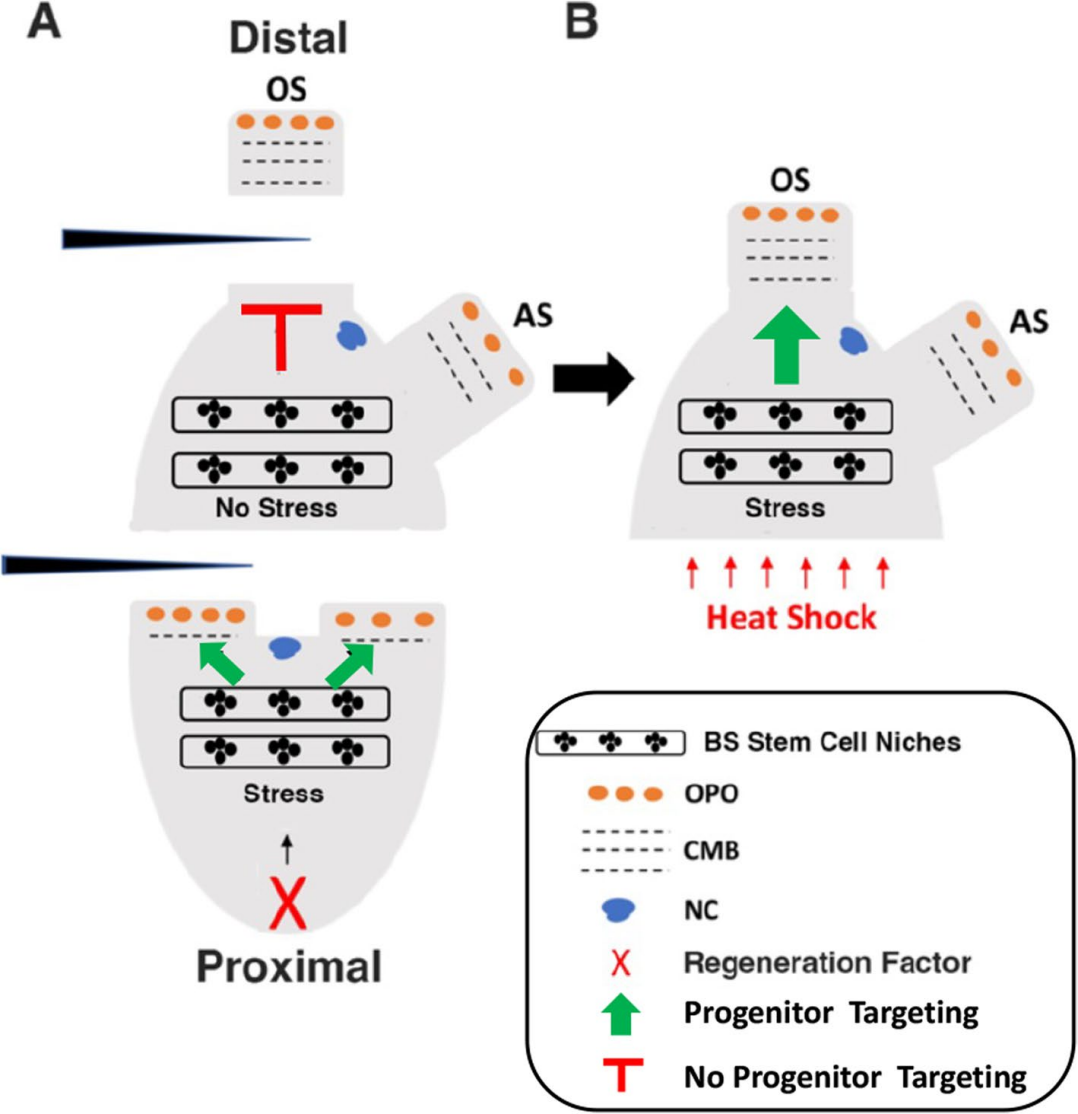
Model for the rescue of oral siphon regeneration by heat shock stress. **A** At normal growth temperature, oral siphon (OS) amputation or bisection into proximal and distal fragments results in the activation of a hypothetical regeneration factor (X) in proximal fragments, which initiates a stress response in the branchial sac vasculature triggering stem cell proliferation, progenitor cell migration, and distal regeneration. Distal fragments without X are unable to activate the stress response to regenerate after OS amputation. **B** A heat shock activates the stress response ectopically in the distal fragment triggering branchial sac stem cell proliferation and OS regeneration

The reason that distal fragments do not mount a stress response after body bisection is still unknown. Based on studies of budding in a colonial tunicate, Berrill and Cohen [55] proposed the existence of a regeneration factor associated with the epicardium, an outgrowth at the base of the proximal pharynx, which would be restricted to proximal fragments after *Ciona* is bisected into two equal parts. Therefore, we suggest a model in which the activation of the HSP70 chaperone system, and/or other heat-inducible systems required for regeneration, is dependent on such a regeneration factor(s), which would activate a BS stress response to trigger regeneration only in the proximal fragments (Fig. 11A). The epicardium or any other proximal visceral organ, including the heart, digestive tract, or gonad, would be a reasonable source of the regeneration factor. According to the model, a heat shock would mimic the proximal regeneration factor and ectopically activate stress and regeneration in distal fragments (Fig. 11B). This model could be tested in future experiments by transplanting different visceral organs into distal fragments and then assaying their capacity for proximal regeneration.

Although complete OS regeneration was accomplished in heat-shocked distal body fragments, they were still unable to reproduce any of the proximal organs, which are known to regenerate from multiple body fragments in *Polycarpa mytiligera* [18]. A simple explanation for the lack of proximal regeneration in *Ciona* distal fragments may be time: we have thus far not been able to keep distal fragments alive in culture for more than about 2 weeks, which may be sufficient to replace the relatively simple OS but not the structurally complicated proximal organs, some of which may contain their own complement of adult stem cells [5, 39, 50]. The appearance of pigmentation around the proximal wound sites in distal fragments (Fig. 10C, D, F, G, N) may be indicative of some progress toward regeneration [6]. Another possibility is that replacement of visceral organs may require the presence of the proposed proximal regeneration factor(s) (Fig. 11A) and/or the specific stem cells located in the proximal organs. Lastly, proximal regeneration could be incompatible with unidirectional polarized growth. *Ciona* has the capacity for distal growth, as evidenced by proximal to distal elongation of nerve and longitudinal muscle fibers [15] and massive distal expansion of the BS during the adult life cycle [19], but may not be capable of growth in a proximal direction. If the latter scenario applies here, then *Ciona* distal regeneration would resemble progressive proximal to distal growth and regeneration in the urodele limb, according to the “rule of distal transformation” [56], and this would imply a deep evolutionary origin of unidirectional growth and regeneration within the chordates.

## Conclusions

This study has identified a large number of DEG in a BS transcriptome, highlighted by the *hsp70*, *dnaJb4*, and *bag3* genes, which may participate in an upregulated HSP70 chaperone system during early *Ciona* regeneration. Thus, a stress response has been revealed that is necessary for the regeneration of distal structures following OS amputation or bisection of the body into proximal and distal fragments, which may function by targeting progenitor cells produced in BS stem cell niches to the sites of distal injuries. Using this information, we have demonstrated that regeneration can be promoted in distal body fragments by a heat shock-mediated stress response. Our results suggest that regeneration is limited in *Ciona* distal body fragments because they do not undergo a stress response.

## Methods

### Biological materials

*Ciona intestinalis* adults were collected in Sandwich Harbor, Cape Cod, USA. Freshly collected adults, about 6 cm in length, were used to prepare BSs for transcriptome analysis. Juveniles were raised from fertilized eggs obtained from adults collected from Sandwich Harbor or raised from eggs dissected from adults grown in the *Ciona* mariculture system at Station Biologique, Roscoff, France. All other experiments used juvenile animals. To produce cultures of juveniles, swimming larvae were allowed to attach and undergo metamorphosis on plastic Petri dishes. The Petri dishes with attached juveniles were placed on racks in aquaria containing unfiltered running seawater, or seawater supplied with algae, maintained at 16 °C and raised to the desired size for operations (1–2 months old with 8–16 transverse vessels in the BS).

### Branchial sac preparation and RNA extraction

The procedure used to obtain BSs for RNA extraction from control and regenerating animals is illustrated in Fig. 1. First, the OS was amputated in 6 recently collected adults of approximately similar size with micro-cautery scissors, as described below. Second, the tunic was removed from the 6 animals with amputated siphons and 3 un-amputated controls by dissection using sharp Watchmaker’s forceps. Third, the BS complex was dissected from each of the 9 animals by cutting longitudinally through the body wall, lifting the branchial tissue from the body wall, severing the connections to the pharynx and the stomach, and immediately submerging in Trizol for RNA extraction. The isolated BS complex contained transverse vasculature, with stem cell niches, the endostyle, and the neural complex, which is connected ventrally to the branchial sac (Fig. 1H). Total RNA was extracted using the RNeasy Lipid Tissue Mini Kit (Qiagen, Valencia, CA, USA) according to the manufacturer’s instructions.

### Transcriptome sequencing and assembly

RNA isolated from the 9 BS samples was used for cDNA library preparation and sequenced using RNA-Seq technology [57, 58], generating 26,331,222 raw sequencing reads and 26,325,012 clean reads after filtering low-quality reads. Bowtie2 [59] was used to map clean reads to reference genes and HISAT [60] was used for mapping to the reference genome. The average mapping ratio to the reference gene was 28.13%, and the average genome mapping ratio was 45.52%. Strict quality control for each sample was used to evaluate the sequencing data.

### Identification of differentially expressed genes

DEGs were identified by DESeq2 [61]. A round number of gene counts from the RNAseq data were used as the input for DESeq2 with one data set containing three BS Day 0, Day 1, and Day 3 replicates. The data set was analyzed in four compared groups: day 1 versus day 0, day 3 versus day 0, day 3 versus day 1, and day 3 versus day 0/1. Genes were filtered with expression values of 0 in at least 78% of the samples. Differential expression data was obtained by comparing each group (fold change > 2; *p*-adjusted < 0.05) with other parameters kept at the default settings. Ensembl transcript IDs were converted to Ensembl gene IDs or gene names by g:Prolifer (https://biit.cs.ut.ee/gprofiler/convert) and DAVID Bioinformatics Resources (https://david.ncifcrf.gov/conversion.jsp).

### Weighted gene co-expression network analysis

To identify gene modules correlated with regeneration in day 3 BS replicates, R-package WGCNA [62] was conducted with the 1149 DEGs identified by DESeq2. The co-expression network was constructed with fragments per kilobase of exon per million mapped fragments (FPKM) using the blockwise modules function for day 3 and day 0/1 BS replicates. An adjacency correlation matrix was calculated for the DEGs, and the correlations were weighted to a soft threshold power β, which favors strong over weak correlations. For each pair of genes, a robust measure of network interconnectedness was calculated based on the adjacency matrix. The parameters used were maximum block size—1000 genes, power (β)—20, minimum module size—25, minimum height for merging modules—0.25, and maximum height for trimming the tree-0.85. The remaining parameters were kept at the default settings.

The co-expression modules associated with day 3 regenerating animals were identified using principal component analysis of gene expression with the blockwise modules function in WGCNA. Each module was summarized by an eigengene, which is the first principal component of the scaled module expression. Thus, the module eigengene explained the maximum amount of variation of the module expression levels. A Student’s asymptotic test with the corP value function was used to determine the *p* values of the correlation.

### Identification of hub genes

Hub genes were identified by their first principal component, the module eigengene, as a summary of overall module expression, using Cytoscape [63] and Network Analyzer [64]. The hub genes were ordered by descending degrees of connectivity, and genes of highest degree were selected to plot the network. The hub genes with lower degrees of connectivity were identified by Cytoscape cytoHubba using the Density of Maximum Neighborhood Component (DMNC) method [65].

### Functional enrichment analysis

GO functional enrichment analysis was conducted using DEG in the WGCNA turquoise and blue modules. The gene function of GO categories was enriched using g:Profiler (https://biit.cs.ut.ee/gprofiler/gost) (FDR < 0.05). The top 20 significant GO term elements in the turquoise and gray modules were visualized by the ggplot2 package in R software.

### Prediction of protein–protein interactions

The Search Tool for the Retrieval of Interacting Genes [66] (STRING, Version 11.00) was employed to predict protein–protein interactions and the results were visualized with Cytoscape. A minimum required interaction score of 0.6 was used, and the remaining parameters were kept at the default settings.

### Oral siphon amputation, body bisection, and heat shock application

Animals were anesthetized by a 15–20-min treatment with 0.6 mg/ml (adults) or 0.2 mg/ml (juveniles) tricaine methane-sulfonate (MS222; Sigma-Aldrich, St. Louis, MO, USA) buffered in Millipore filtered seawater (MFSW). OS amputations were carried out using straight-bladed micro-cautery scissors for wild-captured adults or fine dissection scissors (Fine Science Tools, Foster City, CA, USA) for laboratory-cultured juveniles. OS amputation was done in whole animals or distal body fragments (see below) by a cut along a plane perpendicular to the long axis of the OS at a position immediately below the ring of tentacles, as described previously [15]. It is notable that this operation avoided wounding the underlying BS (Fig. 1H). Bisection of the body was done similarly, but along a plane perpendicular to the proximal–distal axis at the approximate midpoint between the distal and proximal ends, severing the BS into distal and proximal fragments (Fig. 10A). The distal fragments contained the two siphons, the neural complex, the severed distal portion of the BS, the endostyle, and the distal rectum, whereas the proximal fragments contained the proximal portion of the severed BS and endostyle, the proximal rectum, and the underlying viscera. Amputated adults were maintained in running seawater, and amputated juveniles were maintained in MFSW in plastic cell wells, at 16 °C. To apply a heat shock, intact animals, whole distal fragments, or distal fragments with amputated OSs were individually transferred to MFSW (25 °C) for 30 min, and then transferred individually back into 16 °C MFSW for subsequent culture.

### Quantitative reverse transcriptase polymerase chain reaction

RNA was isolated from juveniles using the Direct-zol RNA Microprep kit (Zymo, Irvine, CA, USA). cDNA was synthesized using the SuperScript IV VILO Master Mix and oligo (dT)_20_ primers (Sigma-Aldrich, St. Louis, MO, USA). Quantitative reverse transcriptase PCR (qRT-PCR) was performed using the PCR Master Kit (Roche) under the following cycling conditions: initial denaturation for 2 min at 94 °C, then 35 cycles for 30 s at 94 °C, 30 s for 60 °C, and 1 min for 72 °C, and final extension for 5 min at 72 °C. The primers used for *hsp70* amplification were 5′-GATCTGGGAACAACGTATTCATGC-3′ (forward) and 5′-TGTTTCGTTGAATGCGACATAGC-3′ (reverse), for *dnaJb4* amplification were 5′-CGGATAATGTCTCATGCCAGTTCTC-3′ (forward) and 5′-CCTTTGTATATGTCCTCAAGGGAACAC-3′ (reverse), and for *bag3* amplification were 5′-AAAATGCCTCAACCGTTCGC-3′ (forward) and 5′-CTTTAGGCGCCGCTTGGTTT-3′ (reverse). The primers for amplification of the *rpl11* reference gene were 5′-AGCTGCAAAGGTCTTGGAACA-3′ (forward) and 5′-AACGCACAGTATAGCGAGCC-3′ (reverse). The qRT-PCR reactions were performed using a LightCycler 480 (Roche). The ΔCt for the *hsp70*, *dnaJb4*, and *bag3* genes was calculated by subtracting the average Ct value of the *rpl11* reference gene. To compare gene expression, ΔΔCt was calculated by subtracting the average ΔCt of day 0 from day 3. For graphical representation, the fold change was calculated as 2^−(ΔΔCt)^.

### In situ hybridization

RNA probes were prepared for in situ hybridization by RT-PCR amplification of the *Ciona intestinalis hsp70*, *dnaJb4*, and *bag3* genes using the *hsp70* forward primer 5′-CTCACAGCAGACGGCATTCTAT-3′ and reverse primer 5′-CTTCCGCAGTGTCCTTCATCTT-3′, the *dnaJb4* forward primer 5′-TTTCGGCACTTCAAACCCAT-3′ and reverse primer 5′-ATCAGTACAAGGTAACGGGA-3′, and the *bag3* forward primer 5′-TTTCCCAAACCAAGCGGCG-3′ and reverse primer 5′-TCTTTTTGCGTGCGGCTATG-3′. The PCR products were cloned into the TOPO-TA Dual promoter-cloning vector (Life Technologies). Established procedures for juvenile *Ciona intestinalis* were used for in situ hybridization [21]. Juveniles were relaxed by treatment with MS222 (see above) supplemented with menthol crystals (Sigma-Aldrich, St. Louis, MO, USA) for 30 min, fixed in 4% paraformaldehyde (PFA) in 0.5 M NaCl-MOPS (pH 7.0) buffer overnight at 4 °C, then dehydrated through an increasing ethanol-methanol series, and stored in 100% methanol at − 20 °C. The in situ hybridization and washing procedures were conducted as described [67] with the following modifications: the specimens were incubated in 5 μg/ml Proteinase K dissolved in PBST at 37 °C for 25 min and incubated at 45 °C in hybridization buffer containing 1 ng/μl of antisense DIG-labeled RNA probe. The specimens were stained with BM purple (Roche) and viewed by light microscopy.

### Gene knockdown with short interfering RNA

Gene knockdowns were carried out as described previously [7] using stealth siRNAs designed and synthesized by Invitrogen (Waltham, MA, USA). The sequence of the *hsp70* siRNA was 5′-AGGAAUCGAUCUGGGAACAACGUAU-3′, the sequence of the *dnaJb4* siRNA was 5′-GACCCUCCAAUACAUUGCGAUUUAA-3′, and the sequence of the *bag3* siRNA was 5′-CAGCUCCGCCGGAUUUACGACUUAA-3′. The control stealth siRNA was purchased from Invitrogen. The siRNAs were diluted in RNase-free water, and 100 μM stock solutions were stored at − 20 °C prior to use. The effective siRNA concentrations were determined empirically by assaying the effects of a dilution series on survival and OS regeneration. Juveniles were incubated with 2 μM siRNA in 10-ml volumes of MFSW immediately after OS amputation. At day 3 after OS amputation, the animals were rinsed once with MFSW and incubated in fresh siRNA. The extent of OS regeneration was determined at day 5 after OS amputation by assaying OPO, CMB, and siphon lobe development.

### EdU labeling and quantification

Cell proliferation was determined by incubating siRNA-treated animals and controls with 200 μmoles/L 5′ ethynyl-2′-deoxyuridine (EdU; Invitrogen, Carlsbad, CA, USA) in cell wells (6–7 animals per 10 ml MFSW) for 2 days, followed by a chase consisting of 5 washes with MFSW, and incubation in MFSW for 3 days. EdU pulse and chase-labeled animals were relaxed by treatment with MS222 and menthol crystals for 30 min at room temperature [7] and fixed in 4% PFA for 14 h. The fixed specimens were washed three times in PBS, permeabilized in 0.5% Triton X-100 for 1 h, washed three more times with PBS, processed for EdU detection with Alexa Fluor 488 using the Click-it imagining kit (Thermo Fisher Scientific, Waltham, MA, USA) as described previously [5], and imaged by fluorescence microscopy. EdU-labeled nuclei were quantified by manual counting in 100 μm^2^ areas of amputated OS or BS vasculature (see Fig. 1A) as described previously [7].

### Phalloidin staining

Distal body fragments were fixed in 5% formalin and washed in PBS, and actin filaments were stained with 25 μg/ml rhodamine-phalloidin (Molecular Probes, Eugene, OR, USA) at room temperature in the dark [21]. After staining, the specimens were washed three times in PBS (10 min) and imaged by fluorescence microscopy.

## Supplementary Information

**Additional file 1: Figure S1.** Heatmap of correlation coefficient values across BS replicate samples. Day 0: replicate control BS samples. Day 1: replicate BS samples one day after oral siphon amputation. Day 3: replicate BS samples three days after oral siphon amputation.

**Additional file 2.** Lists of DEGs in Day 1 versus Day 0 BS replicates determined by DESeq2. **Table S1.1.** Upregulated genes. **Table S1.2.** Downregulated genes.

**Additional file 3.** Lists of DEGs in Day 3 versus Day 0/1 BS replicates determined by DESeq2. **Table S2.1.** Upregulated genes. **Table S2.2.** Downregulated genes.

**Additional file 4.** Lists of module genes. **Table 3.1.** Blue module genes. **Table S3.2.** Turquoise module genes. **Table S3.3.** Gray module genes.

**Additional file 5: Figure S2.** GO enrichment analysis of the blue module genes. The number of genes in the top 20 GO terms for Biological Process, Cellular Component, and Molecular functions is shown.

**Additional file 6.** Lists of higher and lower degree of connectivity genes in the turquoise module. **Table 4.1.** Higher degree of connectivity genes. **Table 4.2.** Lower degree of connectivity genes.

**Additional file 7: Table S5.1.** STRING analysis of protein-protein interactions with HSP70.

**Additional file 8: Table S6.1.** Heat shock induction of oral siphon regeneration in distal fragments.

## Data Availability

The datasets used and/or analyzed during the current study are available from the corresponding authors on reasonable request. The transcriptome sequencing data has been deposited in BioProject with National Center for Biotechnical Information (NCBI) under accession number PRJNA953172 [68].

## Abbreviations

AS: Atrial siphon
BS: Branchial sac
BSV: BS vasculature
cDNA: Complementary DNA
CMB: Circular muscle bands
DAVID: Database for Annotation, Visualization, and Integrated Discovery
DEG: Differentially expressed genes
DIG: Dioxygenin
DMNC: Density of Maximum Neighborhood Component
EdU: 5′Ethyny-2′-deoxyuridine
EN: Endostyle
FDR: False discovery rate
GO: Gene Ontology
HISAT: Hierarchical Indexing for Spliced Alignment of Transcripts
HSP: Heat shock protein
LMB: Longitudinal muscle band
MFSW: Millipore-filtered seawater
MS222: Tricaine methyl sulfonate
NC: Neural complex
OPO: Oral siphon pigment organ
OS: Oral siphon
PCR: Polymerase chain reaction
PFA: Paraformaldehyde
PBS: Phosphate-buffered saline
PBST: PBS, 1% Triton X100
qRT-PCR: Quantitative RT-PCR
RT-PCR: Reverse transcriptase PCR
siRNA: Short interfering RNA
STRING: Search Tool for Retrieval of Interacting Proteins
V: Viscera
WGCNA: Weighted Gene Co-expression Network Analysis
