# Supplementary figures and images for: Differentially expressed chaperone genes reveal a stress response required for unidirectional regeneration in the basal chordate Ciona
Source: BMC Biol. 2023 Jun 26;21:148. doi: 10.1186/s12915-023-01633-y (PMC10294541; doi:10.1186/s12915-023-01633-y)

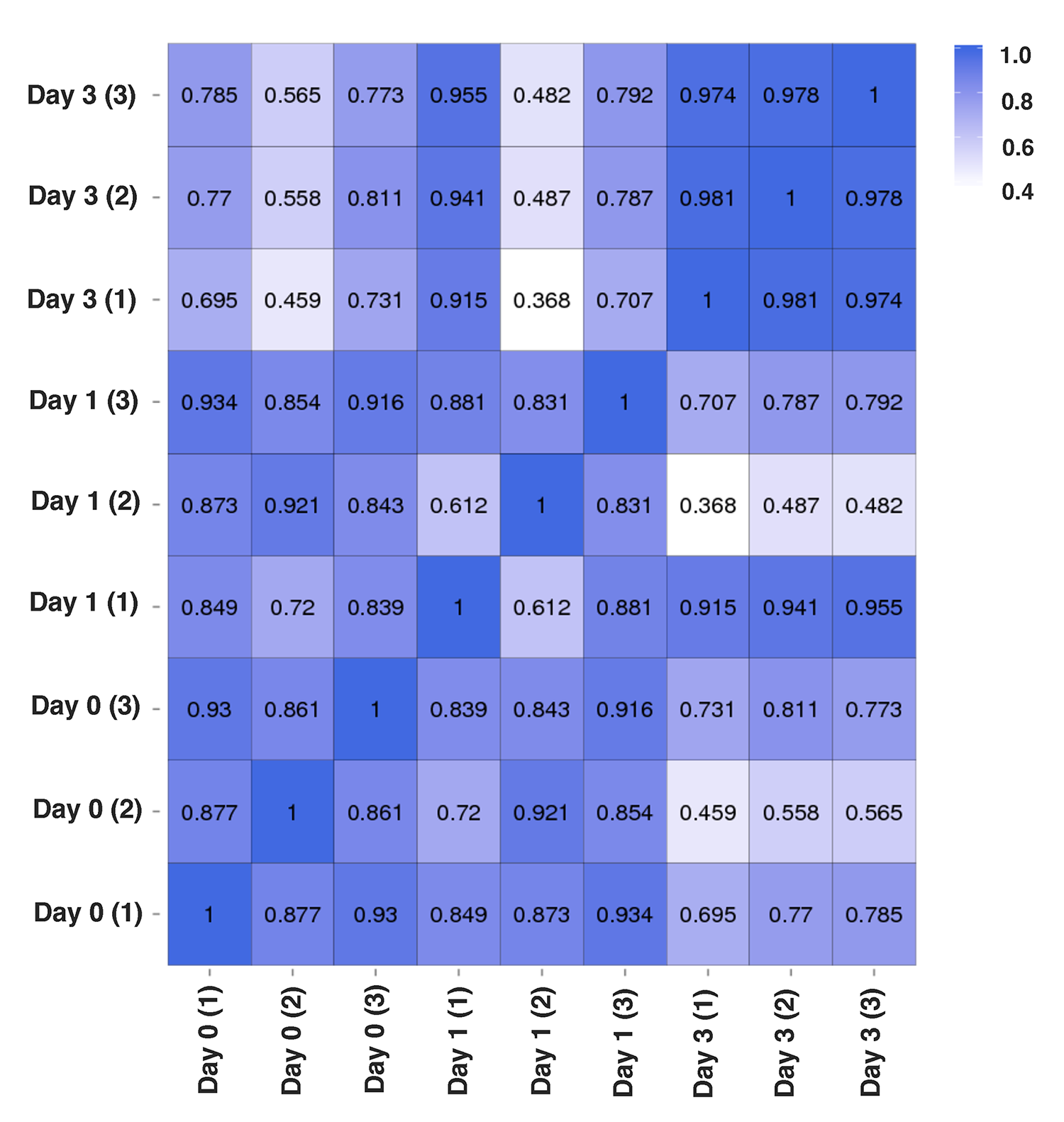

Supplement: Supplementary file 1 — Additional file 1: Figure S1. Heatmap of correlation coefficient values across BS replicate samples. Day 0: replicate control BS samples. Day 1: replicate BS samples one day after oral siphon amputation. Day 3: replicate BS samples three days after oral siphon amputation. [file 12915_2023_1633_MOESM1_ESM.tif]

GO: Biological Process (BP) GO: Cellular Component (CC) GO: Molecular Function (MF)

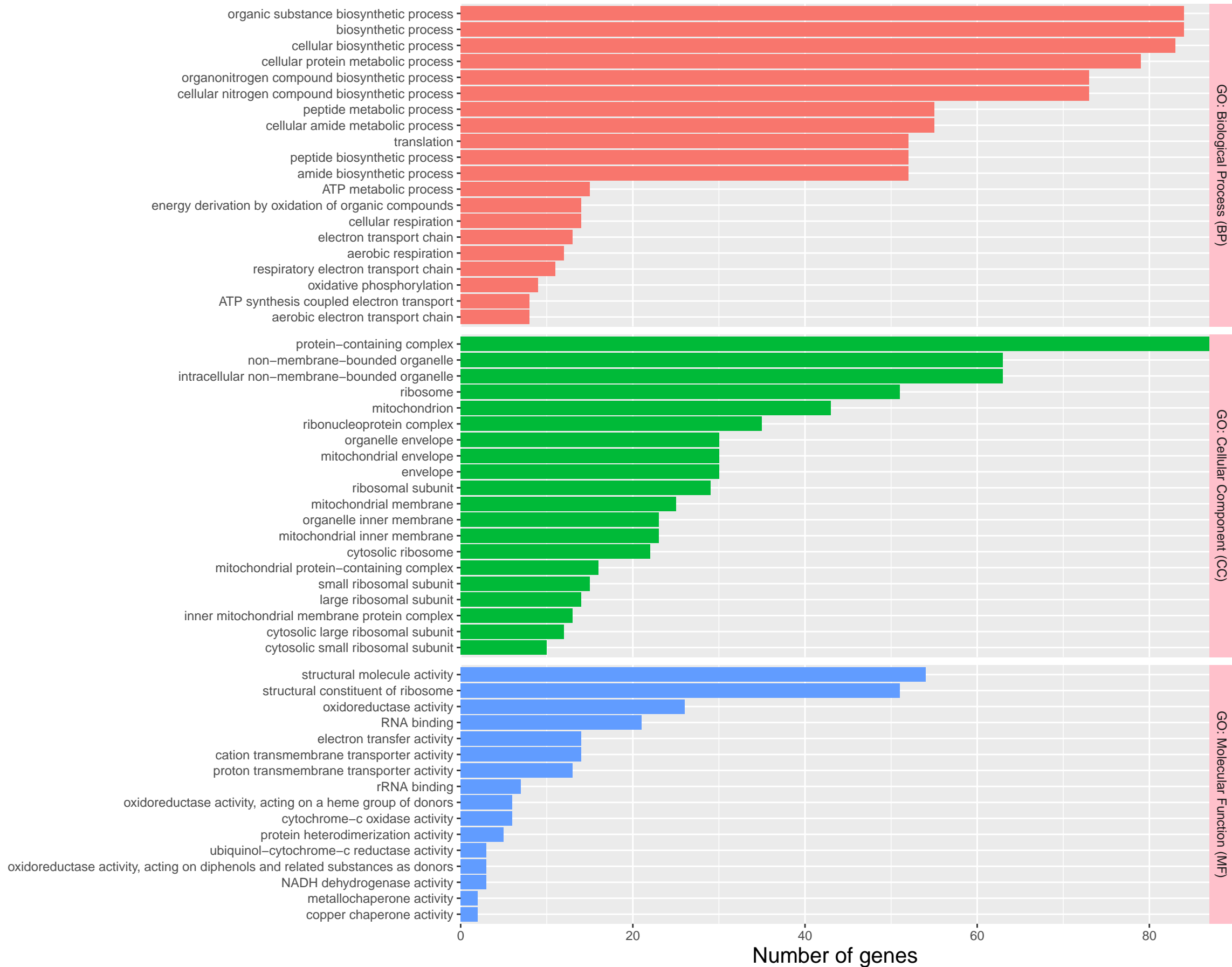

Supplement: Supplementary file 5 — Additional file 5: Figure S2. GO enrichment analysis of the blue module genes. The number of genes in the top 20 GO terms for Biological Process, Cellular Component, and Molecular functions is shown. [file 12915_2023_1633_MOESM5_ESM.pdf]
